# Sorting Reality from What We Think We Know About Breast Cancer in Africa

**DOI:** 10.1371/journal.pmed.1001721

**Published:** 2014-09-09

**Authors:** Sulma I. Mohammed, Joe B. Harford

**Affiliations:** 1 Department of Comparative Pathobiology and Purdue University Center for Cancer Research, Purdue University, West Lafayette, Indiana, United States of America; 2 Center for Global Health, National Cancer Institute, Bethesda, Maryland, United States of America

## Abstract

In a linked Perspective, Sulma Mohammed and Joe Harford discuss barriers to accurate diagnoses of breast cancer in Africa.

*Please see later in the article for the Editors' Summary*

**Linked Research Article**

This Perspective discusses the following new study published in *PLOS Medicine*:

Eng A, McCormack V, dos-Santos-Silva I (2014) Receptor-Defined Subtypes of Breast Cancer in Indigenous Populations in Africa: A Systematic Review and Meta-Analysis. PLoS Med 11(9): e1001720. doi:10.1371/journal.pmed.1001720

In a systematic review and meta-analysis, Isabel dos-Santos-Silva and colleagues estimate the prevalence of receptor-defined subtypes of breast cancer in North Africa and sub-Saharan Africa.

Much attention has been paid to the features of breast cancer in Africa and the parallels between breast cancer in indigenous Africans and in African American women, including a shift toward earlier onset; a tendency toward poorer outcomes; and an increased likelihood for the tumors to be negative for the estrogen receptor (ER), the progesterone receptor (PR), and/or the human epidermal growth factor receptor-2 (HER2) [1],[2]. One of the more aggressive forms of breast cancer is termed “triple negative,” i.e., ER−, PR−, HER2− [3]. Patients with triple negative breast cancer tend to be younger than patients with other forms of the disease, and at all ages, the triple negative subtype is more common in women of African descent than in the white women in the United States [4]–[6].

## Lack of Cancer Research in Africa

A systematic review examining breast cancer subtypes in Africa is published in this week's *PLOS Medicine* by Isabel dos-Santos-Silva and colleagues [7]. A take-home message of the review article is that caution should be exercised in stating what we know (or think we know) about the receptor status of breast cancers in Africa. The paucity of breast cancer–relevant research in Africa means that any generalization will be based on relatively few studies of varying quality from relatively few places on a very diverse continent. Of 54 studies from North Africa, approximately 80% of all the studies reviewed were from Egypt or Tunisia. In sub-Saharan Africa (nearly 50 countries), two countries (Nigeria and South Africa) contributed more than half of the 26 studies. In addition to the limited geographic representation, many studies involved fewer than 300 patients.

## The Asymmetry of Positive and Negative Test Results

dos-Santos-Silva and colleagues [7] note that there has been a substantial increase over time in the proportion of ER+ breast cancers being reported. This increase found in comparing studies prior to 2001 to those after 2007 is more likely a reflection of an increased capability of African pathology laboratories to conduct the tests for receptor status reliably than a true shift in tumor biology. This observation highlights a feature of receptor status determination that is often ignored—namely, that a positive result in a test based on immunohistochemistry is inherently more reliable than a negative result using the same test in the same laboratory. If a sample is somehow mistreated or the test is botched in some way, the test results on even a receptor-positive specimen will be scored as “negative.” In sub-Saharan Africa, those studies employing archival material (tissue blocks) were more likely negative for ER and PR than those based on prospectively analyzed specimens. Similarly, studies from North Africa using formalin-fixed paraffin-embedded blocks reported lower ER+ and PR+ proportions than those using frozen tissue samples, and almost all studies from sub-Saharan Africa utilized formalin-fixed paraffin blocks. Technical factors related to specimen characteristics, including preparation and storage, would tend to bias results asymmetrically toward receptor negativity and lead to overestimates of receptor negativity relative to the true underlying biology of the tumors.

Routine testing for receptor status remains uncommon in most of sub-Saharan Africa. When such tests are attempted, potential issues that may lead to poor results include overdilution of the formalin to stretch the reagent and poor control of timing and conditions of fixation, e.g., poor buffering of pH. In some venues, tissue samples are actually given to patients in formalin who keep the specimens at home (sometimes for months, with temperatures greater than 100°F) until they can afford the cost of the receptor status test(s). In some settings where ER status is unknown and tamoxifen is available and affordable, all patients with breast cancer are given tamoxifen even though only ER+ patients would be expected to benefit [8]. Health system policy regarding cost and resource availability may also come into play. It is our understanding, for example, that the National Health Insurance System in Ghana covers the cost of tamoxifen for breast cancer patients but does not cover the cost of the test for ER status.

Another very important point made by dos-Santos-Silva and colleagues [7] is that expressing receptor status in simple terms of the proportion of breast cancers that are ER− may be of limited usefulness. Two populations with identical incidence rates of ER− breast cancer may have different proportions for this form of breast cancer if total breast cancer rates differ in the two populations. Proportions of certain forms of breast cancer (e.g., inflammatory, triple negative, or in males) are often cited as being higher in Africa without acknowledging that these higher proportions are actually being driven mathematically by lower rates of other forms of breast cancer rather than higher rates of the form in question [9].

## The Age Structure of the African Population

The mean age for breast cancer in Africa is undeniably younger, but one must remember that the mean age for almost anything in Africa is likely to be younger, because Africa has by far the youngest population of any continent on the planet [10]. Given that age is the single most substantive risk factor for most cancers, including breast cancer, a younger population will have both lower overall incidence of breast cancer and a lower mean age of onset based simply on the demographics of the population. This point has been made previously in comparing the relatively younger population of Egypt with that of the US [11], but all of the principles of this comparison would apply to the younger populations that exist throughout Africa and, indeed, in other younger populations, e.g., in Arab populations [12]. It should also be noted that the median age of diagnosis of non-Hispanic black women in California was reported to be 7 years younger than that of non-Hispanic white women covered by the same cancer registry [9]. The likelihood of a US woman having a triple negative tumor is highest in premenopausal black women [5]. In Africa, a much larger fraction of the breast cancer cases happens to be in premenopausal black women, because they make up a much larger fraction of the population there. The question has been raised as to whether Africans are more likely to be diagnosed at a younger age because they are triple negative or if they are more likely to be triple negative because they are younger [3]. It should also be noted that a propensity toward negative receptor status is by no means limited to Africans and African American women. In a review of data from the Surveillance, Epidemiology, and End Results (SEER) Program, racial or ethnic groups in the US that had elevated risk of diagnosis with ER−/PR− breast cancers relative to non-Hispanic white women included Native Americans, Filipinos, Chinese, Koreans, Vietnamese, Indians/Pakistanis, Mexicans, South or Central Americans, and Puerto Ricans living in the US, in addition to African Americans [13].

The systematic review of dos-Santos-Silva and colleagues [7] serves to caution against overstating what we know (or think we know) about breast cancer in Africa. The authors suggest that the distribution of receptor-defined subtypes of breast cancer in Africa may not be dramatically different from that found in Western populations, given the younger age overall of the population in Africa. We would concur with the authors that much more research is needed to sort what we believe to be true from the reality of breast cancer in Africa.
